# The Interplay Between Non-Instantaneous Dynamics of mRNA and Bounded Extrinsic Stochastic Perturbations for a Self-Enhancing Transcription Factor

**DOI:** 10.3390/e28020238

**Published:** 2026-02-19

**Authors:** Lorenzo Cabriel, Giulio Caravagna, Sebastiano de Franciscis, Fabio Anselmi, Alberto D’Onofrio

**Affiliations:** 1 Department of Mathematics, Informatics and Geosciences, University of Trieste, Via Economo 12/3, 34132 Trieste, Italy; 2 Instituto de Astrofísica de Andalucía, Consejo Superior de Investigaciones Científicas (IAA-CSIC), Glorieta de la Astronomía s/n, 18008 Granada, Spain

**Keywords:** mRNA, bounded noises, extrinsic stochasticity, phase transitions, Sine–Wiener noise, delay differential equations

## Abstract

In this work, we consider a simple bistable motif constituted by a self-enhancing Transcription Factor (TF) and its mRNA with non-instantaneous dynamics. In particular, we mainly numerically investigated the impact of bounded stochastic perturbations of Sine–Wiener type affecting the degradation rate/binding rate constant of the TF on the phase-like transitions of the system. We show that the intrinsic exponential delay in the TF positive feedback, due to the presence of a mRNA with slow dynamics, deeply affects the above-mentioned transitions for long but finite times. We also show that, in the case of more complex delays in the feedback and/or in the translation process, the impact of the extrinsic stochasticity is further amplified. We also briefly investigate the power-law behavior (PLB) of the averaged energy spectrum of the TF by showing that, in some cases, the PLB is simply due to the filtering nature of the motif. A similar analysis can also be applied to biological models having a qualitatively similar structure, such as the well-known Capasso and Paveri–Fontana model of cholera spreading.

## 1. Introduction

One of the most important fields of theoretical biophysics is the so-called systems biology [1,2,3], a discipline that has been mainly created by Ilya Prigogine and that has the invaluable merit of having introduced into modern biochemistry (and chemistry in general) and molecular biology a factor previously, and rather surprisingly, neglected: time. Before Prigogine, time only mattered in the phase from out of equilibrium to equilibrium. After Prigogine’s introduction, in macroscopic and mesoscopic worlds, of the concept of breaking of time and space symmetry, the meaning of equilibrium definitely changed [4,5,6]. Another major advance in statistical biophysics is due to two strict collaborators of Prigogine: Werner Horsthemke and Ren’e Lefever, who first showed that stochasticity may have constructive effects—termed Noise-Induced Transitions—in nonlinear physical systems [7,8], including in chemistry and biochemistry [9]. Indeed, they showed that Gaussian noise perturbing mono-stable nonlinear systems may induce multi-modal stationary probability densities. In cell biochemistry, protein levels are often associated with cellular functions, which implies that NITs relate to the emergence of functional biological heterogeneity in changing environments [10]. This is in perfect coherence with the concept of emergent properties via the emergence of dissipative structures, again due to Prigogine [6,11].

The quantitative dynamic studies of biochemical systems are primarily focused on protein dynamics, and in particular on the dynamics of Transcription Factors. Therefore, the majority of models neglect the role of mRNAs by assuming that they are in a quasi-steady state [1,2,3]. This, for example, is the approach that three of us (GC, SdF, AdO) have followed in [10,12,13], where the impact of extrinsic stochasticity on the dynamics of proteins has been assessed. In particular, in [10], it has been considered, from a statistical mechanics point of view, the perturbations of the protein decay/binding rate constant (DBRC) in a circuit modeling the positive feedback of a transcription factor (TF) on its own synthesis, which was investigated first quantitatively in a deterministic framework in [14]. The DBRC models both the spontaneous degradation of the TF and its linkage to other unknown biomolecular factors or drugs. However, the characteristic evolution time of mRNAs is often comparable to the characteristic evolution times of the protein, which implies that one cannot consider the dynamics of mRNA at a quasi-steady state [1,2,3]. To start, in 2001, Raghavan and colleagues [15] performed a genome-wide analysis of mRNA decay in resting and activated primary human T-lymphocytes and found that the half-life of investigated mRNAs was often larger than 30 min; for example, IL-4 ($t_{1/2}\approx 144$ min), p56 ($t_{1/2}\approx 240$ min), IL-2 (in both cases, the respective half-times range between 23 and 150 min), and many others. In 2010, Lee and colleagues [16] investigated mouse C2C12 myoblasts and discovered that a number of mRNAs have very long decay times. Namely, they showed that the median decay time is 2.9 h, i.e., 174 min, that 90% of mRNAs have a mean decay time longer than 96 min, and that in 10% of cases, the decay time is longer than 300 min.This is not surprising since, in the fundamental work [17], Sharova et al. investigated the mRNA half-life of 19,977 genes obtained via DNA microarray analysis of pluripotent and differentiating mouse embryonic stem cells, obtaining important results. For example, in that work, when investigating the stability of mRNA in mouse ES cells, they showed that, in certain differentiation-related conditions, a substantial percentage of mRNAs have a half-life longer than 24 h and that the histogram of frequencies of half-times has a flat mode with equal frequencies between 5 h and 7.5 h. A specific example is *Oct4*, whose protein half-life is roughly 12 h and whose mRNA half-life is approximately 7.5 h [18]. As a consequence, we may say that, in a number of relevant biological scenarios, the mRNA dynamics must not be neglected both in deterministic [1,2,3] and stochastic [19,20] modeling. As first shown in the sixties by Goodwin [21] and Griffith [22,23], this induces effects of interest in many scenarios investigated in a number of extremely important works, among which we mention: [1,2,3,19,20,21,22,23,24,25,26,27,28,29,30,31,32,33].

In particular, two key studies [19,20] investigate the interplay between intrinsic stochasticity and a realistic non-instantaneous dynamics of mRNA in the above-mentioned basic auto-regulatory network with positive feedback.

Due to the extreme relevance of the dynamics of mRNA in scenarios where it cannot be considered at a quasi-steady state with respect to the dynamics of the associated TF, in this work, we propose a novel model of the effect of extrinsic bounded stochastic perturbations affecting the dynamics of a self-enhancing Transcription Factor and its mRNA via bounded stochastic perturbations of the TF degradation rate.

The simple motif we investigate here has great theoretical relevance as an intrinsic generator of bistability [14,20,34,35,36,37,38,39], a phenomenon of paramount relevance in many key intracellular mechanisms; for example, in governing cell fate decision [40]. The simple motif under study has been experimentally observed in some biomolecular networks; For example, (i) in human marrow stromal cell differentiation in response to BMP2 protein stimulation [41]; (ii) in the ComK TF network of *B. subtilis* [42,43]; (iii) in the developmental decision pathway of bacteriophage λ [44]; (iv) in many pathways related to the phenotypic regulation of bacteria [43]; (v) in TATA Binding Protein, also known as TBP [45]; (vi) in the galactose GAL3 signaling switch in yeast cells [46]; (vii) in both plants and bacteria, especially in *E. coli*, there are various examples of Response Regulators interacting with sensor histidine kinase (HK) that self-activate their production [36,37,47,48]. Moreover, the tet-Off system can lead to an equivalent model [49]. Passing to the increasingly important domain of synthetic biology, the paradigm of a TF with self-positive feedback has been employed to design a pioneering synthetic eukaryotic gene switch in *Saccharomyces cerevisiae* by Becskei and coworkers in 2001 [50], and more recently [51].

We model the fluctuations of the degradation rate (due, e.g., to stochastic binding to other chemicals) as bounded stochastic fluctuations, which are inherently colored, rather than by means of Gaussian white or colored noises (GWCNs). Indeed, modeling random perturbations of intrinsically positive and limited parameters of linear or nonlinear models using classical GWCNs induces gross biological artifacts [10,12,13,52,53] owing to the unboundedness of the Gaussian distribution [10,12,13,52]. From a statistical physics perspective, it is currently accepted that noise sources should preserve the positivity and boundedness of perturbed parameters [52,54,55], a viewpoint that has generated a large body of literature [56,57,58,59,60,61,62,63,64,65,66,67,68,69,70,71,72,73,74]. These biophysical considerations directly apply to the scenario investigated here.

This manuscript is organized as follows: in Section 2, we provide key information on inferring the deterministic dynamic model of the TF-mRNA pair; Section 3 is devoted to a simple analytical study on how bounded stochastic perturbations afflicting parameter *d* depend on the decay rate of the TF δ; in Section 4, we provide and illustrate the results of the performed numerical simulations; Section 5 proposes an extended stochastic model that takes into the account the non-instantaneous nature of both the cellular feedback and of the protein translation; concluding remarks ends this work, but are followed by Appendix A containing the results of the simulations in Section 5.

## 2. The Deterministic Dynamics of TF-mRNA: Biological and Systems Biology Details

The model adopted here, which extends the one introduced by Smolen, Baxter and Byrne in [14], represents a single transcription factor that self-activates. In [14], Smolen and colleagues relate their model to several real gene networks involving homodimer TFs, such as activating protein-1 (AP-1), a family of Fos–Jun heterodimers that bind to phorbol ester–responsive elements. Responsive elements that bind TFs have indeed been shown to affect the transcription of genes encoding diverse TFs; for example, Jun autoregulates its own transcription.

More precisely, in [14], it is assumed that: (i) the gene producing the TF has only one promoter (although the presence of multiple TF binding sites would lead to a similar deterministic model as in [48]); (ii) the active form of the TF is its homodimer; (iii) the homodimerization rate is very fast, so that the homodimer remains in equilibrium with the TF and the homodimerization process is not explicitly represented in the model; (iv) gene activation is triggered by binding to the homodimer; (v) even in the absence of binding, there is a small baseline activation rate, independent of gene binding to homodimers. In addition to the assumptions made in [14], we further assume that: (vi) the dynamics of the mRNA are not fast with respect to the dynamics of the TF and therefore cannot be neglected.

In this work, as in [1,2,3,14], we suppose that both the TF, denoted as *P*, and the mRNA, denoted as *M*, are sufficiently abundant to be modeled by differential equations.

Regarding the switching dynamics of gene activation/deactivation, we assume, as in [3,20,22,23], that the activation propensity is enhanced by binding to the homodimeric form of the TF, so that:

$$Prob\left(G\left(t+dt\right)=1|G\left(t\right)=0\right)=\left({\bar{c}}_{0}+{\bar{c}}_{2}P^{2}\right)dt$$

$$Prob\left(G\left(t+dt\right)=0|G\left(t\right)=1\right)={\bar{b}}_{0}dt,$$

where G(t) denotes the activation state of the gene associated with the TF. Namely: (i) G(t)=0 corresponds to the gene being OFF; (ii) G(t)=1 corresponds to the gene being ON.

Adopting a differential model implies that we consider the case in which the rate constants are large and comparable. Thus, they can be rewritten as

$$\left({\bar{b}}_{0},{\bar{c}}_{0},{\bar{c}}_{2}\right)={\bar{c}}_{0}\left(\beta_{0},1,{\hat{\gamma }}_{2}\right)$$

if ${\bar{c}}_{0}\gg 1$ then, at the timescales of both the mRNA and TF dynamics, we may approximate

(1) $$G\left(t\right)\approx \langle G\left(t\right)\rangle =\frac{1+{\hat{\gamma }}_{2}P^{2}}{\beta_{0}+1+{\hat{\gamma }}_{2}P^{2}}$$

In this way, denoting time by τ, it follows that

(2) $$\begin{array}{cc} \frac{dM}{d\tau } & =k_{0}n\frac{1+{\hat{\gamma }}_{2}P^{2}}{\beta_{0}+1+{\hat{\gamma }}_{2}P^{2}}-\delta_{0}M \end{array}$$

(3) $$\begin{array}{cc} \frac{dP}{d\tau } & =\theta_{0}M-d_{0}P \end{array}$$

where $k_{0}$ denotes the transcription rate for large *y*, $\delta_{0}$ the degradation rate of the mRNA, $\theta_{0}$ the translation rate, and $d_{0}$ the degradation rate of the TF. The parameter *n* represents the number of gene copies. In this work, we consider the diploid case, in which the TF is produced by two copies of *G* (n=2). However, in pathological conditions, either fewer or more gene copies may be required: (i) due to heterozygous gene loss, n=1 [75]; (ii) in tumor cells, n>2 owing to polyploidy [76]. We assume that all gene copies have practically identical kinetics, a standard hypothesis in biochemical kinetics [20,34]. The resulting coupled ODE system (2)–(3), derived from approximation (1), describes a regime in which the dynamics are governed by fast gene switching and a high abundance of proteins [13]. We adimensionalize time with respect to the characteristic protein timescale and set

$$t=d_{0}\tau ,\;M=\eta x,\;P=\xi y.$$

It is straightforward to verify that, if one chooses

$$\eta =\frac{k_{0}n}{\delta_{0}},\;\xi =\theta_{0}\eta ,\;{\hat{\gamma }}_{2}\xi^{2}=\gamma_{2},$$

and sets $\delta =\delta_{0}/d_{0}$ and d=1, one obtains:

(4) $$\begin{array}{cc} \frac{dx}{dt} & =\delta \left(\frac{1+\gamma_{2}y^{2}}{\beta_{0}+1+\gamma_{2}y^{2}}-x\right) \end{array}$$

(5) $$\begin{array}{cc} \frac{dy}{dt} & =x-dy \end{array}$$

For δ≫1, applying the quasi-steady-state approximation yields the model [14]:

(6) $$\frac{dy}{dt}=\frac{1+\gamma_{2}y^{2}}{\beta_{0}+1+\gamma_{2}y^{2}}-dy$$

Models (4) and (5) inherit key properties from model (6). For example, if $y_{e}$ is an equilibrium of (6), then $z_{e}=\left(dy_{e},y_{e}\right)$ is clearly an equilibrium of (4) and (5). Moreover, the system (4)–(5) is cooperative; hence, Kamke’s theorem [77] on differential inequalities applies.

## 3. Bounded Stochastic Perturbations Afflicting the Parameter d: Definitions, Simple Analytical Inferences and Sine–Wiener Bounded Noise

In this section, we investigate the interplay between the finite degradation rate constant δ of the mRNA and extrinsic stochastic fluctuations modeled as bounded noisy oscillations of the parameter *d*, namely the degradation/binding rate constant of the TF under study. Under this assumption, the following bi-dimensional random differential system is obtained:

(7) $$\begin{array}{cc} \frac{dx}{dt} & =\delta \left(\frac{1+\gamma_{2}y^{2}}{\beta_{0}+1+\gamma_{2}y^{2}}-x\right) \end{array}$$

(8) $$\begin{array}{cc} \frac{dy}{dt} & =x-d(1+\nu (t))y \end{array}$$

where ν(t) is a bounded stochastic process satisfying

$$\langle \nu \left(t\right)\rangle =0;\;B_{\max}\geq \nu \left(t\right)\geq B_{\min}>-1\Rightarrow 1+\nu \left(t\right)>0.$$

For δ≫1, the quasi-steady-state approximation yields

$$x\approx \frac{1+\gamma_{2}y^{2}}{\beta_{0}+1+\gamma_{2}y^{2}},$$

so that the dynamics reduce to the scalar ODE

$$\frac{dy}{dt}=\frac{1+\gamma_{2}y^{2}}{\beta_{0}+1+\gamma_{2}y^{2}}-d\;\left(1+\nu \left(t\right)\right)\;y,$$

previously investigated in [10]. For simplicity, we set

ν(t)=Bξ(t),

where ξ(t) is a symmetric bounded noise with ξ(t)∈[−1,1].

Setting $z\left(t\right)=\left(x(t),y(t)\right)$, we obtain

(9) $$z^{\prime }=F\left(z,B\xi \left(t\right)\right),$$

which is cooperative since

$$\partial_{z_{2}}F_{1}>0,\;\partial_{z_{1}}F_{2}>0.$$

Hence, since

(10) $$F\left(z,B\right)<z^{\prime }<F\left(z,-B\right),$$

and by cooperativity of the model, it follows that

(11) $$z_{B}\left(t\right)<z\left(t\right)<z_{-B}\left(t\right).$$

Here,

(12) $$\frac{d}{dt}z_{U}\left(t\right)=F\left(z_{U},U\right),\;z_{U}\left(0\right)=z\left(0\right).$$

The properties of the generic system (12) are therefore of interest. We first consider the equilibrium points, which are of the form $\left(y_{e}\left(U\right),y_{e}\left(U\right)\right)$, where $y_{e}\left(U\right)$ solves

$$d\left(1+U\right)=\frac{1}{y}\frac{1+\gamma_{2}y^{2}}{\beta_{0}+1+\gamma_{2}y^{2}}.$$

We focus on the case in which exactly three solutions exist, which requires

$$d_{m}<d\left(1+U\right)<d_{M}.$$

Thus, in this case, we have three solutions:

$$y_{L}\left(U\right),y_{c}\left(u\right),y_{R}\left(U\right)\;\mathrm{with}\;y_{c}\left(u\right)\in \left(y_{L}\left(U\right),y_{R}\left(U\right)\right).$$

It is an easy matter to verify that the three corresponding equilibria are such that

- $E_{L}\left(U\right)=\left(y_{L}\left(U\right),y_{L}\left(U\right)\right)$ is Locally Asymptotically Stable (LAS)
- $E_{C}\left(U\right)=\left(y_{C}\left(U\right),y_{C}\left(U\right)\right)$ is unstable
- $E_{R}\left(U\right)=\left(y_{R}\left(U\right),y_{R}\left(U\right)\right)$ is Locally Asymptotically Stable (LAS)

Less trivial is to note that the set

$$A_{U}={\left[0,y_{C}\left(U\right)\right)}^{2}$$

is positively invariant. Therefore, if $z_{U}\left(0\right)\in A_{U}$, then

$$z_{U}\left(t\right)\to E_{L}\left(U\right),$$

i.e., $E_{L}\left(U\right)$ is globally attractive in $A_{U}$. Indeed, it is

div(F(z;U))=−δ−d<0.

Similarly, the set:

$$G_{U}={\left(y_{C}\left(U\right),+\infty \right)}^{2}$$

is positively invariant, so that if $z_{U}\left(0\right)\in G_{U}$ then

$$z_{U}\left(t\right)\to E_{R}\left(U\right).$$

Hence, since (11) holds, if $z\left(0\right)\in A_{-B}\subset A_{B}$ then

$$z\left(t\right)\in L={\left[y_{L}\left(B\right),y_{L}\left(-B\right)\right]}^{2}.$$

Similarly, if $z\left(0\right)\in G_{B}\subset G_{-B}$ then, for sufficiently large times,

$$z\left(t\right)\in R={\left[y_{R}\left(B\right),y_{R}\left(-B\right)\right]}^{2}.$$

This implies that whenever

$$d_{m}<d\left(1+U\right)<d_{M}$$

the stochastic model admits (*independently from the details of the perturbing bounded noise*!) at least two distinct stochastic attractors. Indeed, denoting by ρ(x,y,t) the PDF of the random vector (x,y) and setting

$$\Omega =Supp\left(\rho (x,y,0)\right),$$

if $\Omega \subset A_{-B}$, then, for large times,

$$Supp\left(\rho (x,y,t)\right)\subset L,$$

whereas if $\Omega \subset G_{B},$ then, for large times,

$$Supp\left(\rho (x,y,t)\right)\subset R.$$

**Remark** **1.** *The above analytical investigation also applies to general bistable models of the type*

(13) $$\begin{array}{cc} \frac{d}{dt}X_{1} & =a\left(R\left(X_{2}\right)-X_{1}\right) \end{array}$$

(14) $$\begin{array}{cc} \frac{d}{dt}X_{2} & =X_{1}-X_{2} \end{array}$$

*sharing the same qualitative properties as our model and perturbed in a similar way by bounded noise. For example, the multistability model of the lactose utilization network in E. coli proposed by Ozbudak and coworkers [78], which focuses on the interplay between TMG and LacY, is of type (13)–(14). Moving to the epidemiology of infectious diseases, our findings can also be applied to the well-known cholera spreading model of Capasso and Paveri-Fontana [79]. The only difference with our case is that in the cholera model [79], R(0)=0 and $R^{\prime }\left(0\right)>0$, which simply implies that $y_{L}\left(U\right)=0$ and $d_{m}=R^{\prime }\left(0\right)$.*

In the next sections, we numerically investigate the impact of the noise size *B*. To carry out this investigation, however, we must specify both the model of the bounded noise employed and the numerical values of the parameters.

### Bounded Stochastic Processes

Temporal bounded noises (BNs) can be modeled either by applying a suitable bounded transformation to an unbounded stochastic process or by means of dedicated stochastic differential models [52].

Regarding the first class of BNs, in this work, we consider one of the most widely used processes, namely the so-called Sine–Wiener noise [52,59,60,80,81,82]. This important bounded noise was independently introduced in [80,82] as a particular case of random perturbations of the phases of harmonic phenomena:

$$a\left(t\right)=\sin\left(\omega t+\sqrt{\frac{2}{\tau }W\left(t\right)}\right)$$

Setting ω=0 yields the Sine–Wiener noise: [52,59,60,81,82]

$$\xi \left(t\right)=\sin\left(\sqrt{\frac{2}{\tau }W\left(t\right)}\right).$$

The term Sine–Wiener was most likely first introduced by Bobryk and Chrzeszczyk in [60].

The stationary PDF of ξ(t) is given by [52,59,60,81]

$$P_{\infty }\left(\xi \right)=\frac{A}{\sqrt{1-\xi^{2}}}$$

It can be shown [55,60] that, for sufficiently large times, the autocorrelation function of the SW BN is of Ornstein–Uhlenbeck type,

$$R_{\xi \xi }\left(\theta \right)=\frac{1}{2}\exp\left(-\frac{\left|\theta \right|}{\tau }\right)$$

so that the associated power spectrum reads [83]

$$\phi_{\xi \xi }\left(\omega \right)=\frac{1}{\pi \tau }\frac{1}{\omega^{2}+\frac{4}{\tau^{2}}}.$$

## 4. Base Model Simulations

The aim of this study is to determine whether the more realistic framework in which the mRNA dynamics are explicitly modeled, leading to a bi-dimensional cooperative system, modifies the scenario reported in [10]: a first-order ‘phase transition’ at a critical noise amplitude $B_{1}$, followed by a second-order transition. In particular, we investigate the role of the mRNA degradation rate δ, a new key parameter to be taken into account.

With these considerations in mind, we study the impact of δ on the state variables x(t) and y(t) for different values of *B*. In a plot of x(t) and y(t) similar to that proposed in [10], for each value of *B*, we generated 1000 trajectories and recorded their values at T=50,000.

For both x(t) and y(t), we plotted the mean value of the trajectories together with their minimum and maximum values.

In all cases, the system was perturbed by a Sine–Wiener bounded noise with τ=10.0. The other parameters were chosen as follows [10]:

$$\gamma_{2}=65.536;\;\beta_{0}=15.0;\;d=1.0;$$

Regarding δ, we considered the following values:

δ∈{2.5,0.5,0.3,0.1}

All simulations were performed in C++ using our SDE-SS [84] library.

**Figure 1:**
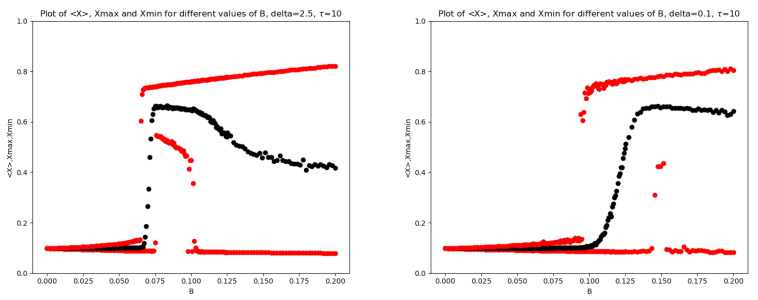
Base model: plots of 〈X〉 (black), $X_{max},X_{min}$ (red) vs. *B*. (**Left** panel) δ=2.5; (**Right** panel) δ=0.1.

**Figure 2:**
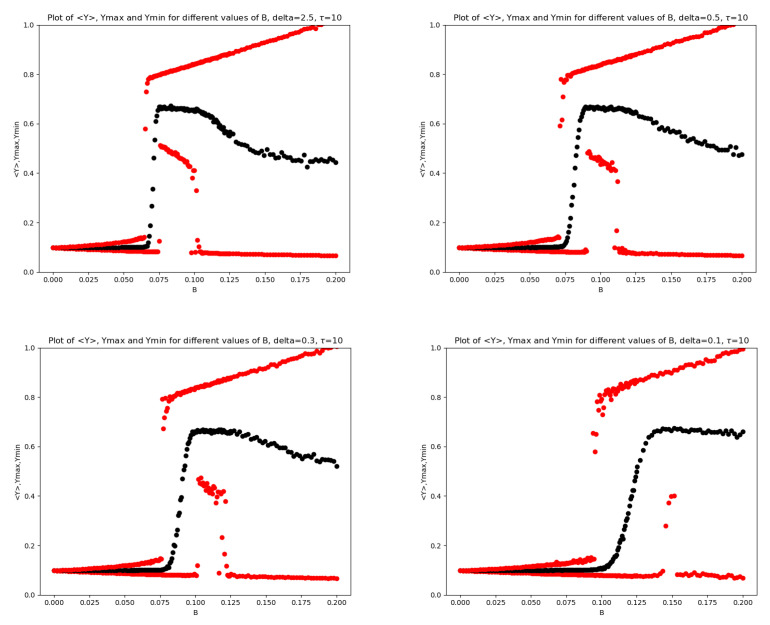
Plots of 〈Y〉 (black), $Y_{max},Y_{min}$ (red) for different values of *B* for the base model. On the (**top left**), δ=2.5, on the (**top right**) is δ=0.5, on the (**bottom left**) is δ=0.3 and on the (**bottom right**) is δ=0.1.

In Figure 1, we report the mean (together with max and min) of x(T) as a function of *B* for two values of the mRNA-degradation rate: δ=2.5 and δ=0.1. In Figure 2, the analogous plots are shown for the Transcription Factor for all four values of δ.

In the left panel of Figure 1 and in the upper left panel of Figure 2, both corresponding to δ=2.5, a scenario slightly richer than that reported in [10] can be observed. In particular, three characteristic values of the noise amplitude can be identified, namely $B_{0}$, $B_{1}$, and $B_{2}$, such that: (i) at $B=B_{0}$, the maximum value of the noisy oscillations abruptly jumps to a large level; (ii) at $B=B_{1}$, a noise-induced irreversible transition to small oscillations around a large mean value occurs through a sudden increase in the minimum value of the stochastic oscillations; (iii) at $B=B_{2}$, a second-order transition takes place, characterized by a sudden decrease in the minimum value of the oscillations, which cease to be small and become large.

In contrast, in the right panel of Figure 1 and in the lower right panel of Figure 2, both corresponding to δ=0.1, radically different dynamics are observed. The maximum value increases abruptly, the average value increases gradually, and the minimum value remains small. The intermediate region has almost completely disappeared.

**Figure 3:**
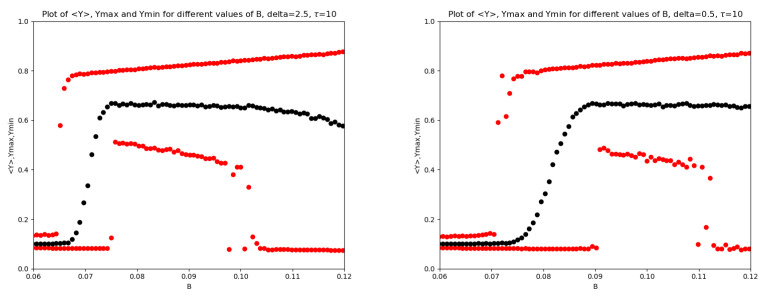
Zooms of the plots of 〈Y〉 (black), $Y_{max},Y_{min}$ (red) for different values of *B* for the base model. On the (**left**), δ=2.5, on the (**right**) is δ=0.5.

In the remaining two panels of Figure 2, a decrease in δ produces a rightward shift of the intermediate region, together with a visible reduction of its width and a progressive disappearance of this portion of the plot. Moreover, the transition region around $B_{1}$ becomes increasingly elongated as δ decreases; a similar behavior is observed in the descending branch on the right-hand side of the plots. In particular, in the transition between the first and the second region, the system evolves from a sudden and sharp jump to an almost sigmoid transition behavior. To better highlight this transition region, we also report, for δ=2.5 and δ=0.5, a zoomed view of the *Y* variable in Figure 3.

**Figure 4:**
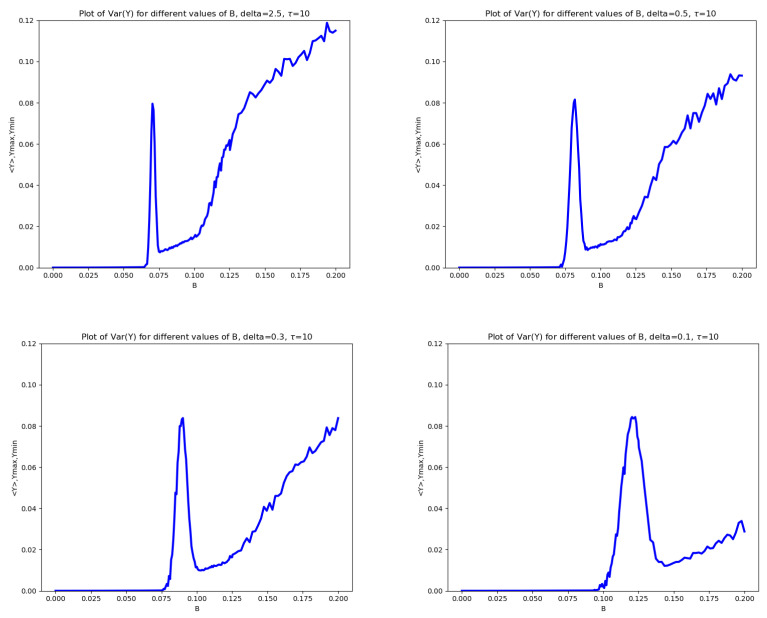
Variance of Y(T) versus *B* for the base model. On the (**top left**), δ=2.5, on the (**top right**) is δ=0.5, on the (**bottom left**) is δ=0.3 and on the (**bottom right**) is δ=0.1. In all panels, τ=10.

The transitions illustrated above in the key statistics of both the TF and mRNA concentrations are accompanied by significant changes in their variances. In this section, we focus on the dependence of the variance of y(T) on the noise amplitude *B*. Figure 4 reports the variance–*B* plots corresponding to the same pairs (δ,τ) considered in Figure 2.

A pronounced peak in the variance appears in the middle of the transition between the first and the second regime. After this peak, the variance drops abruptly, in correspondence with the transition toward higher values of the minimum of y(T). Subsequently, as soon as $\min\left(y(T)\right)$ decreases, the variance increases owing to the enlargement of the domain explored by y(T). This peak is particularly meaningful, as it reveals a highly ambiguous and unpredictable behavior of the system for the *B* values belonging to the transient region. In particular, this behavior is associated with the non-guaranteed transition of trajectories from the first regime to the second during the system’s evolution.

Across all the tests performed, and also in the subsequent ones, the behavior of *x* is very similar to that of *y*; the only difference is that the upper red line is usually less steep. Moreover, for values larger than δ=2.5, the base model shows almost no differences in either *x* or *y*. A similar situation occurs between 2.5 and 0.5, where a smooth transition connects the regime with higher δ to that characterized by lower values.

### 4.1. Spectral Behavior

In this section, we analyze the properties of the averaged Energy Spectrum of the non-dimensional protein concentration, interpreted as the output of a nonlinear filter whose input is the noise. Note that, unlike standard linear filters [85] and most nonlinear filtering frameworks [85], the input here is not additive. Instead, it interacts nonlinearly with the filter through the term ν(t)y.

**Figure 5:**
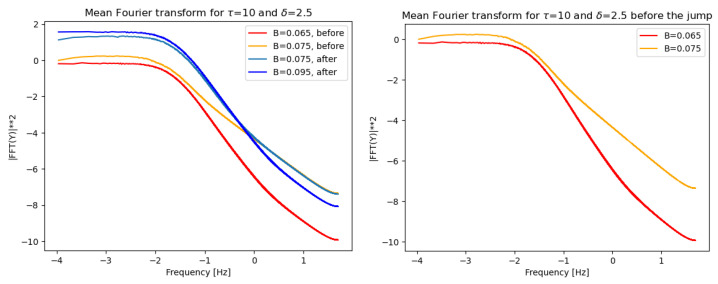
Normalized mean Fourier Transform of time series, in log–log scale for values of the noise amplitude *B* corresponding to the scenarios immediately before and immediately after the “jump” between the first and second region (**left** panel). (**Right** panel) Zoom on two values of *B*. Parameters: δ=2.5 and τ=10 and four values of *B*.

Figure 5 shows a log–log plot of the normalized mean (over 1000 realizations) of the FFT Energy Spectrum (i.e., the squared Fourier transform) of the time series for noise amplitudes slightly below or above the critical amplitude. For the trajectories with B=0.0075, two sets of time series were generated because trajectories in this region initially evolve in the “before the jump” regime and subsequently move toward the upper values of the second regime. Inspection of the transforms reveals that (i) after an initial plateau, a central frequency range exhibits linear behavior, i.e., the FT follows a power law; (ii) at larger frequencies, the plot displays a slower decay, indicating that the FT of the time series does not transition to a behavior that decays faster than a power law.

In our case, the emergence of power-law behavior admits a simple explanation. Observing that the time series corresponding to B=0.65 exhibits a small but non-negligible spread around a mean value $y_{m}$, it follows (and we verified numerically) that $x\approx R(y_{m})$. We therefore consider the following approximate first-order filter with nonlinear input

$$\dot{y}\approx R\left(y_{m}\right)-\left(1+B\sin\left(cW(t)\right)\right)y$$

Setting $y=R\left(y_{m}\right)+R\left(y_{m}\right)BV$, we obtain

(15) $$\dot{V}=-\sin\left(cW(t)\right)-\left(1+B\sin\left(cW(t)\right)\right)V$$

which represents a linear filter with one additive input ($-\sin\left(cW(t)\right)$) and one multiplicative input ($B\sin\left(cW(t)\right)V\left(t\right)$). To disentangle the respective roles of linear versus nonlinear filtering and of the parameter *B*, we performed simulations of the energy spectra for: (i) the nonlinear filtering; (ii) the linear filter of Equation (15) with B=0.065; (iii) the linear filter of Equation (15) with additive input and very small multiplicative input with B≈0.0001; (iv) the linear filter of Equation (15) with maximum multiplicative input, i.e., B=0.9999. In all cases, the Energy Spectra were fitted with the following piecewise linear curves featuring three ‘knees’:

(16) $$Log_{10}\left(ES\right)=A-\sum_{j=1}^{3}b_{j}ReLU\left(Log_{10}\left(f\right)-Log_{10}\left(f_{j}\right)\right),$$

where ReLU(q)=Max(q,0), $f_{j}$ denote the thresholds separating different power-law regimes and

$$f_{1}<f_{2}<f_{3}.$$

Exponentiating both sides of (16) yields

$$ES\left(f\right)=e^{A}Min\left(1,{\left(\frac{f}{f_{1}}\right)}^{-b_{1}}\right)Min\left(1,{\left(\frac{f}{f_{2}}\right)}^{-b_{2}}\right)Min\left(1,{\left(\frac{f}{f_{3}}\right)}^{-b_{3}}\right)$$

(however, if $b_{j}<0$ then one has to replace Min with Max).

We fit the function defined in (16) to the Energy Spectrum using a simple strategy: (i) move the knee points over a grid; (ii) perform a minimum least-squares fit with respect to the remaining parameters; (iii) adopt the following stopping criterion: ‘within a prescribed maximum number of iterations, select the knees that minimize the residuals of the Minimum Least-Squares fit of the remaining parameters’. For the sake of clarity, we stress that the above algorithm is by no means a novel contribution of this work. Rather, it can be viewed as a simplified version of well-established, more sophisticated algorithms for piecewise linear data fitting; see [86,87] and the references therein.

**Figure 6:**
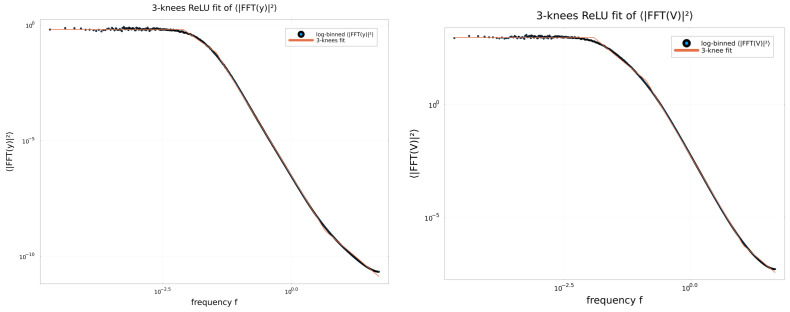

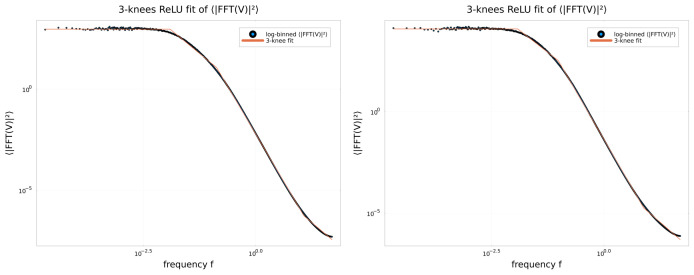
Filtering effects inducing power law behavior as shown by Averaged Energy Spectrum (Square of the FFT of the out of transitory time series) and three kneed approximating piecewise linear fitting curves. (**Upper left** panel) ES of the output of the nonlinear model for B=0.065; (**Upper right** panel) ES of the output of the ***linear*** filter for B=0.065; (**Lower left** panel) ES of the output of the ***linear*** filter for B=0.0001; (**Lower right** panel) ES of the output of the ***linear*** filter for B=0.9999. In all panels: τ=10, T=50,000. In the **upper left** panel, δ=2.5.

Both the computed Energy Spectra and their piecewise linear approximations are shown in Figure 6. The corresponding fitting results are summarized in Table 1 and indicate that: (i) the power-law behavior arises from the filtering nature of the system; (ii) the contribution of the linear filter is dominant; (iii) the ES linear filter depends only weakly on the noise amplitude *B*.

### 4.2. Impact of the Noise Autocorrelation

**Figure 7:**
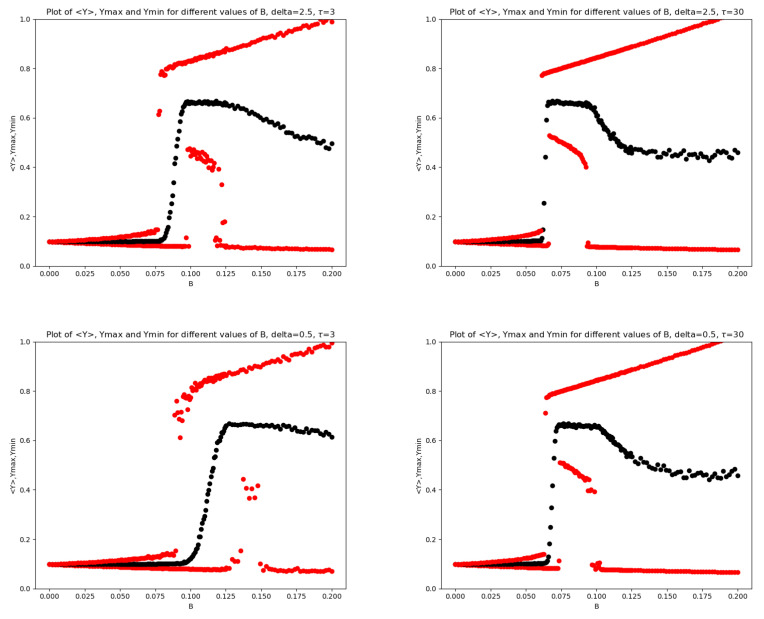

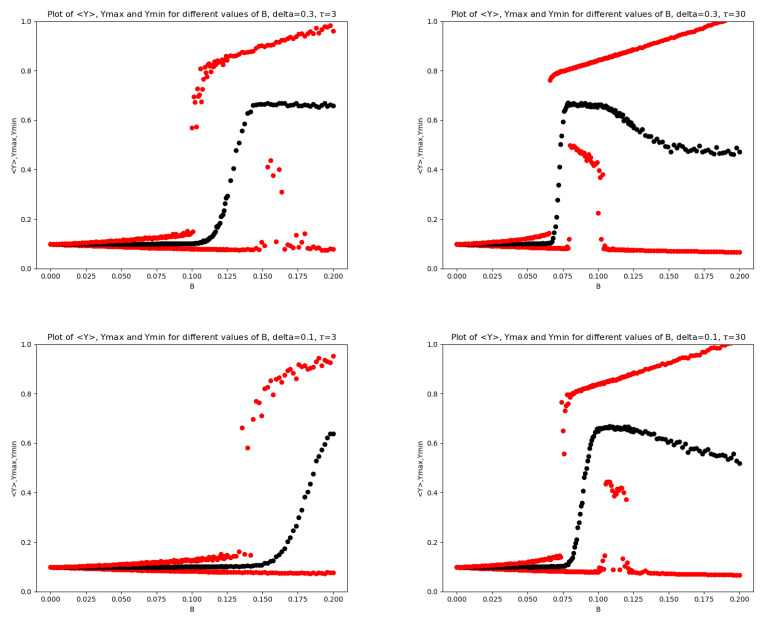
Impact of the bounded noise autocorrelation τ. Plots of 〈Y〉 (black), $Y_{max},Y_{min}$ (red) versus *B* for τ=3 (**left** panels) and for τ=30 (**right** panels). (**First row** panels) δ=2.5; (**second row** panels) δ=0.5; (**third row** panels) δ=0.3; (**lower** panels) δ=0.1.

Regarding the impact of the noise autocorrelation constant τ on protein and mRNA dynamics, Figure 7 compares the scenarios for τ=3 (left panels) and τ=30 (right panels). The plot indicates that for small τ, the transition is either weak or disappears altogether (within the simulated range of noise amplitudes), whereas larger values of τ enhance the sharpness of the transitions.

### 4.3. Probability Distributions

**Figure 8:**
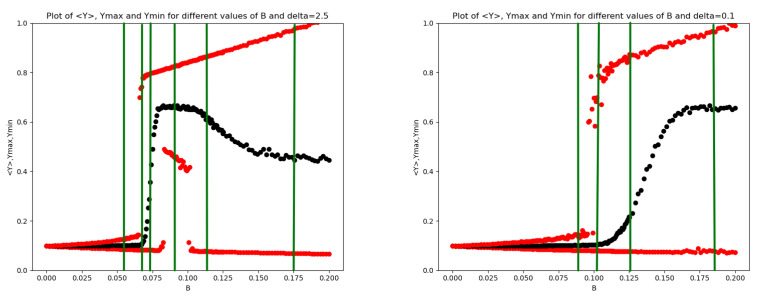
Base model: plots of 〈Y〉 (black), $Y_{max},Y_{min}$ (red) vs. *B*. (**Left** panel) δ=2.5; (**Right** panel) δ=0.1. In green, the *B* values used for the PDF are highlighted.

In this section, we analyze the full distribution of y(T) for selected pairs (B,δ). Regarding the mRNA degradation rate, we consider δ=2.5, characterized by the presence of an intermediate region with large minimum oscillation values, and δ=0.10, characterized by low minimum oscillation values and a gradual decrease in the average. The values of *B*—listed below and shown in Figure 8—have been chosen to explore the main behavioral regimes of the model described in the previous section:

δ=2.5:B∈{0.055,0.065,0.07,0.085,0.115,0.175};δ=0.1:B∈{0.08,0.105,0.125,0.18}

**Figure 9:**
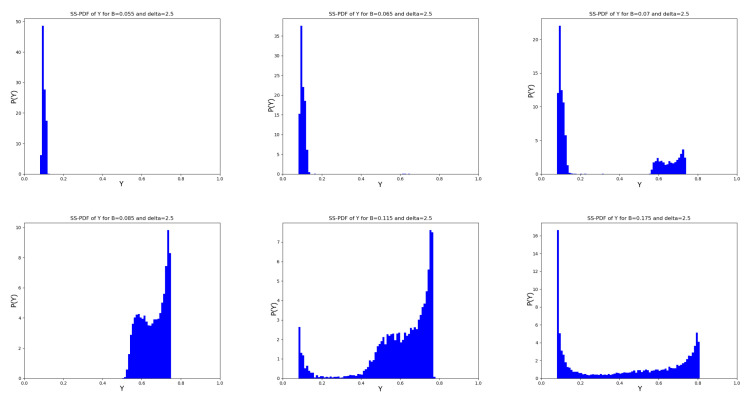
Noise amplitude modulation of the steady state PDF of y(T) for δ=2.5. (**Upper left** panel) B=0.055; (**Upper central** panel) B=0.065; (**upper right** panel) B=0.07; (**Lower right** panel) B=0.085; (**Lower central** panel) B=0.115. (**Lower right** panel) B=0.175.

We begin with the case δ=2.5 by examining the six panels shown in Figure 9. The upper left panel displays the PDF corresponding to B=0.055, i.e., well before $B_{0}$; accordingly, the distribution is concentrated around the lower attractor and exhibits only small oscillations. For B=0.065 (upper central panel), the transition toward large maximum oscillations has just occurred: the mean value remains small, and the PDF is still centered around the lower attractor, although some non-zero bins already appear at large values of y(T). The upper right panel shows a three-modal distribution, characterized by a broad peak at low values of y(T) and two smaller modes at higher values. The lower left panel corresponds to the region where the sudden transition of the minimum has taken place; the PDF becomes bimodal and concentrated within a bounded region at large values of y(T). In the remaining lower panels, we observe the ‘rebirth’ of the lower peak together with a three-modal structure of the PDF. In the lower central panel, the dominant modes correspond to large TF values, whereas the opposite behavior is observed in the lower right panel.

**Figure 10:**
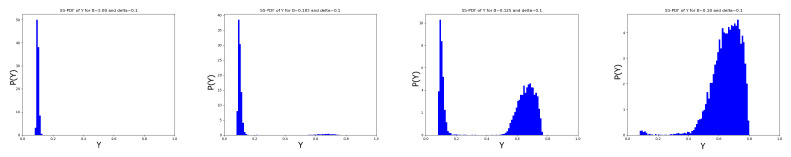
Not normalized SS-PDF of y(t) for δ=0.1. (**First** panel) B=0.08; (**Second** panel) B=0.105; (**Third** panel) B=0.125. (**Fourth** panel) B=0.18.

We now consider the case δ=0.1 and report in Figure 10 the PDFs corresponding to the four values of *B* indicated in the right panel of Figure 8.

Some differences arise, although several features remain common with the case δ=2.5. Once again, for low values of *B*, the PDF is concentrated around the lower attractor. As *B* increases up to 0.105 (first and second panels), the distribution becomes comparable to that obtained for (B,δ)=(0.065,2.5), with almost all simulations located on the left side but with a few non-zero bins appearing on the right, reflecting the similar position at the onset of the transition. Beyond this point, however, the differences dominate: the absence of the intermediate region for δ=0.1 leads directly to a trimodal distribution, characterized by one low mode and two modes at large TF values. For B=0.18, the increase in the mean value causes the support of the PDF to be essentially concentrated at large TF values.

## 5. The Interplay of the Extrinsic Noise with Non-Instantaneous Feedback and/or Non-Immediate Translation

Since the seminal work of Goodwin [21], it has been recognized that the assumptions of instantaneous translation and instantaneous feedback to the nucleus represent strong approximations. Translation, in fact, may occur over non-negligible time intervals. Moreover, the feedback mechanisms that enhance or suppress gene activity require the translocation of the TF into the nucleus, a process that itself takes a finite amount of time.

We therefore extended our model to incorporate both of these effects, obtaining the following stochastic integro-differential system (to be complemented by the model of the bounded noise ν(t))

(17) $$\begin{array}{cc} \frac{dx}{dt} & =\delta \left(\frac{1+\gamma_{2}z^{2}}{\beta_{0}+1+\gamma_{2}z^{2}}-x\right) \end{array}$$

(18) $$\begin{array}{cc} \frac{dy}{dt} & =w-(d+\nu (t))y \end{array}$$

(19) $$\begin{array}{cc} w(t) & =\int_{0}^{\infty }K_{x}\left(s\right)x\left(t-s\right)ds \end{array}$$

(20) $$\begin{array}{cc} z(t) & =\int_{0}^{\infty }K_{y}\left(s\right)y\left(t-s\right)ds \end{array}$$

where w(t) and z(t) denote delayed versions of the adimensional mRNA and TF variables, respectively. The delay kernels satisfy

$$\int_{0}^{\infty }K_{j}\left(s\right)\;ds=1$$

for j=x,y and can be interpreted as the PDFs of the corresponding delays.

Note that: (i) if $K_{x}\left(s\right)=K_{y}\left(s\right)=\delta \left(s\right)$, the baseline non-delayed model is recovered; (ii) setting only $K_{y}\left(s\right)=\delta \left(s\right)$ yields a model in which only the feedback delay is present; (iii) setting only $K_{x}\left(s\right)=\delta \left(s\right)$ yields a model in which translation is non-instantaneous.

We conducted simulations for these three cases in order to compare their behavior with that of the baseline model. The results, reported in detail in Appendix A, indicate that, within the simulated scenarios, the delay does not substantially alter the stochastic dynamics of the mRNA–Protein pair subject to extrinsic stochastic fluctuations of the protein decay rate.

## 6. Concluding Remarks

In this work, we investigated the impact of bounded stochastic fluctuations in the degradation rate on the dynamics of a prototypical self-enhancing Transcription Factor (TF) motif and its associated mRNA. After formulating the model, we first carried out an analytical investigation of the proposed stochastic system. This was possible because, in the presence of positive feedback (as in our case), mRNA–TF systems are cooperative [77], i.e., they belong to the class of monotone ODE systems. From this analysis, we showed that, under feasible conditions, the system may admit at least two distinct stochastic attractors whose coexistence collapses as the noise amplitude varies. Moreover, transitions may occur from a stochastic attractor characterized by a low maximum value to another with a large minimum value, a hallmark of phase transition phenomena.

We then performed extensive numerical simulations over a very large but finite time horizon (final time T=50,000). As for the type of bounded noise, we adopted the well-known Sine–Wiener noise [52,59,60,80,81,82].

We found that the intrinsic delay induced by non-instantaneous mRNA dynamics has significant consequences with respect to the Smolen–Baxter–Byrne model with stochastically varying degradation rate [10]. Our analysis suggests that, in similar contexts, the identification of first- or second-order transitions is more effectively achieved by focusing on the behavior of the minimum of the PDF rather than on the average values of the variables. In our model, the average value displays trends that do not faithfully represent the behavior of the full distribution. Summarizing the effects of the intrinsic delay: (i) increasing the noise amplitude beyond a first threshold leads to a smooth increase in the average value; (ii) the minimum value of the protein (mRNA) exhibits a sudden jump, indicating a first-order-like phase transition occurring well after the first critical noise level; (iii) increasing the average lifetime of the mRNA progressively shortens and eventually suppresses the interval in which this first-order transition appears.

We also carried out a spectral analysis of the model. The Spectral Energy of the TF time series (averaged over 1000 realizations) exhibits a power-law behavior; however, this should not be interpreted as evidence of criticality. By fitting the Energy Spectrum with a piecewise linear function, we showed that: (i) the observed power law can be interpreted as the response of the system acting as a nonlinear filter with multiplicative Sine–Wiener stochastic input; (ii) the dominant contribution arises from linear filtering; (iii) within this linear-filtering regime, the power-law exponent depends only weakly on the noise amplitude.

Finally, we extended the model to include non-instantaneous translation and delayed self-feedback of the TF. The simulations indicate that, across all scenarios considered, the stochastic dynamics of the mRNA–TF pair remain qualitatively similar to that described above (see Appendix A).

Note that all the above results can be extended to similar bistable systems featuring exponential delays in the positive feedback and analogous bounded stochastic perturbations driven by Sine–Wiener noise. As already mentioned in Section 3, our analysis naturally extends to the Capasso and Paveri–Fontana model of cholera spread [79,88].

Of course, our work has several limitations. First, a more realistic description could incorporate the discrete nature of both mRNA and TF populations, as done for the adiabatic TF-only scenario of [13]. Second, extrinsic stochastic fluctuations may affect additional biochemical processes [13]. Third, alternative classes of bounded stochastic processes could have been considered [13,52,54,83]. Finally, the fitting of the piecewise linear function to the Energy Spectrum could have been performed using a global optimization algorithm.

Finally, we note that over the last sixty years a large number of seminal works [1,2,3,19,20,21,22,24,25,26,27,28,29,30,31,32,33] (a non-exhaustive and admittedly biased list) have inspired an impressive body of subsequent research. We believe that this extensive literature urgently deserves a comprehensive review highlighting the importance of explicitly accounting for mRNA dynamics, and we hope that the present work may encourage colleagues to undertake such a review, which would be valuable both scientifically and pedagogically.

## Figures and Tables

**Table 1:** 3-knee ReLU fit summary (rounded). $f_{j}=10^{L_{j}}$, $m_{2}=b_{1}+b_{2}$, $m_{3}=b_{1}+b_{2}+b_{3}$.

Case	*B*	*A*	$f_{1}$	$b_{1}$	$f_{2}$	$b_{2}$	$m_{2}$	$f_{3}$	$b_{3}$	$m_{3}$
Lin	0.0001	2.97	0.0124	1.84	0.14	1.94	3.78	11.55	−1.88	1.90
Nonl	0.065	−0.19	0.008	1.56	0.035	2.09	3.65	4.49	−1.75	1.9
Lin	0.065	3.26	0.012	1.67	0.099	1.996	3.66	5.21	−1.79	1.88
Lin	0.9999	4.08	0.012	1.73	0.095	2.09	3.82	7.65	−1.899	1.92

**Figure A1:**
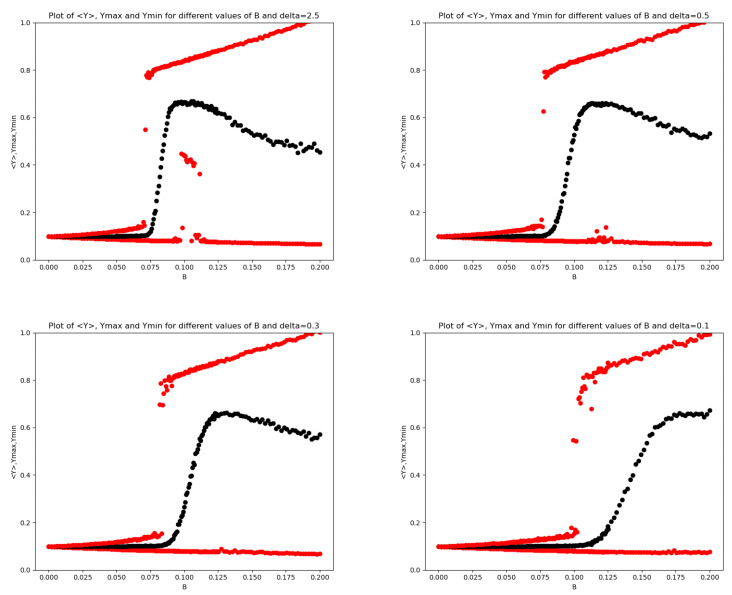
Delayed feedback on the TF: plots of 〈Y〉 (black), $Y_{max},Y_{min}$ (red) vs *B*. (**Top left**) δ=2.5. (**Top right**) δ=0.5. (**Bottom left**) δ=0.3. (**Bottom right**) δ=0.1.

**Figure A2:**
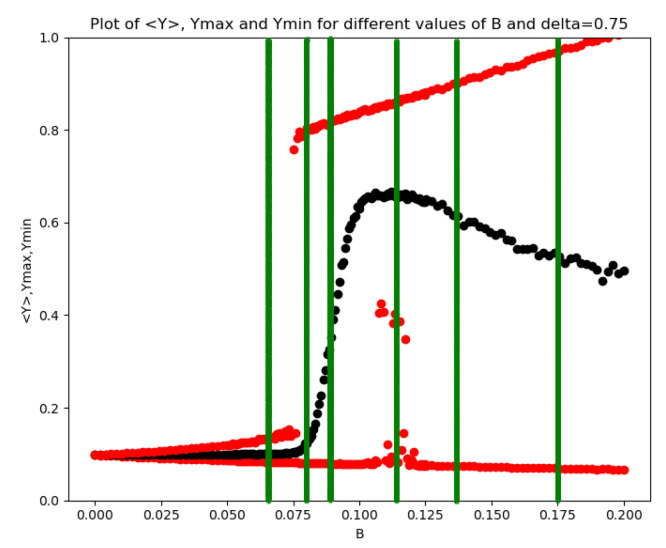
Base model: plots of 〈Y〉 (black), $Y_{max},Y_{min}$ (red) vs. *B* with δ=0.75. In green, the *B* values used for the PDF are highlighted.

**Figure A3:**
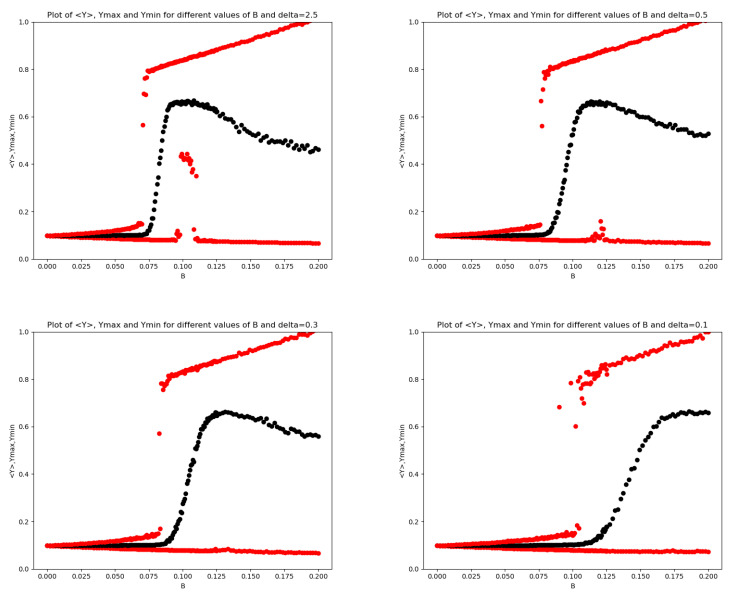
Non-instantaneous translation: plots of 〈Y〉 (black), $Y_{max},Y_{min}$ (red) vs *B*. (**Top left**) δ=2.5. (**Top right**) δ=0.5. (**Bottom left**) δ=0.3. (**Bottom right**) δ=0.1.

**Figure A4:**
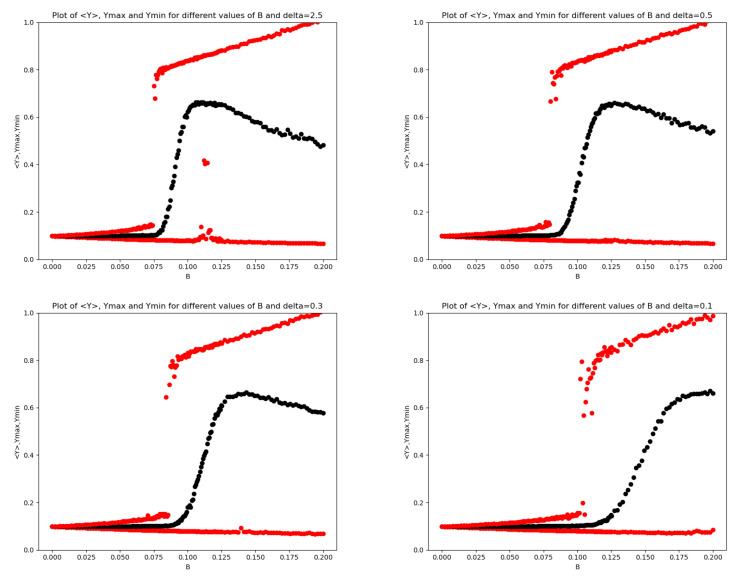
Two delays combined: plots of 〈Y〉 (black), $Y_{max},Y_{min}$ (red) vs *B*. (**Top left**) δ=2.5. (**Top right**) δ=0.5. (**Bottom left**) δ=0.3. (**Bottom right**) δ=0.1.

## Data Availability

The simulated data are available from the authors upon reasonable request.

## Abbreviations

The following abbreviations are used in this manuscript:

TF	Transcription Factor
mRNA	messenger Rybo-Nucleic Acid
FT	Fourier Transform
FFT	Fast Fourier Transform

## Appendix A. The Role of the Non-Instantaneous Nature of Both the Translation and the Feedback Enacted by the TF

### Appendix A.1. Delayed Feedback of the TF

Models assuming instantaneous TF feedback on its own production represent an idealized situation that is seldom realized in practice, since the regulatory effect typically involves a delay associated with TF translocation into the nucleus.

Within our framework, this corresponds to the following stochastic delay- differential system:

(A1) $$\begin{array}{cc} \frac{dx}{dt} & =\delta \left(\frac{1+\gamma_{2}z^{2}}{\beta_{0}+1+\gamma_{2}z^{2}}-x\right) \end{array}$$

(A2) $$\begin{array}{cc} \frac{dy}{dt} & =x-(d+\nu (t))y \end{array}$$

(A3) $$\begin{array}{cc} z(t) & =\int_{0}^{\infty }K_{y}\left(s\right)y\left(t-s\right)\;ds \end{array}$$

where z(t) denotes a delayed version of y(t) generated through the kernel $K_{y}\left(t\right)$.

Assuming an acquisition-fading kernel, the system admits a finite- dimensional representation:

(A4) $$\begin{array}{cc} \frac{dz_{1}}{dt} & =a_{1}\left(y-z_{1}\right) \end{array}$$

(A5) $$\begin{array}{cc} \frac{dz}{dt} & =a_{2}\left(z_{1}-z\right) \end{array}$$

For this variant of the base model, we performed numerical simulations to compare its behavior with that of the original system. In particular, we analyze the quantities $\langle Y\rangle ,Y_{max},Y_{min}$, as shown in Figure 2. As before, we omit the discussion of x(T) because its behavior is qualitatively similar. The simulations were carried out for the same values of δ considered previously, with the aim of highlighting possible differences.

Regarding parameter choices, the common parameters were set equal to those used in the base-model simulations. Suitable values for $a_{1}$ and $a_{2}$ were then selected by fixing the characteristic delay time to $\tau_{c}=1$, which yields the relation $a_{1}a_{2}=a_{1}+a_{2}$. To preserve simplicity and avoid introducing bias, we imposed $a_{1}=a_{2}$, leading to $a_{1}=a_{2}=2$. Throughout this Appendix and the following ones, the simulations are performed over a time horizon T=10,000.

The results for the model with delayed TF feedback are reported in Figure A1. Examining the plots for the different values of δ, we can identify both differences and common features. Beginning with the similarities, the overall behaviors remain qualitatively close to those of the base model: no major structural changes or bifurcations are observed, indicating that the introduction of the delay does not drastically alter the dynamics. The most relevant effect, visible in all four cases, concerns the structure of the intermediate region. Although δ takes the same value in the corresponding panels, the delayed model appears to anticipate the effects associated with decreasing δ, particularly in the width and position of the central region. This effect is especially evident in the top-right panel where, compared with Figure 2, the residual traces of the intermediate region disappear completely. In the delayed setting, the case δ=0.5 resembles more closely the δ=0.3 scenario of the base model. Hence, the presence of delayed TF feedback effectively mimics a slightly smaller δ in the base model.

As in the base model, we now examine the PDFs for the same reference values. The case δ=0.1 remains almost indistinguishable from that of the base model, and thus the PDFs obtained for identical *B* values are highly comparable. The most interesting situation arises for δ=0.75. As observed in Figure A1, this delayed variant appears to anticipate the behavior of the system at lower effective values of δ relative to the base model. Aside from this anticipatory shift, however, no qualitative differences emerge when suitable δ values are chosen in the two models to represent comparable dynamical regimes. Consequently, adopting the same *B* values used for the base-model PDFs would lead to different distributions because the transition regions have shifted. For this reason, we repositioned the six reference values used in the base model, as illustrated in Figure A2.

The selected *B* values are {0.07,0.078,0.09,0.115,0.135,0.175}. Despite repositioning the sampling points, the resulting PDFs exhibit almost no significant differences in their distributions.

We may therefore conclude that, for the parameter ranges considered, introducing delayed TF feedback primarily reinforces the effect of decreasing δ: larger δ values in the delayed model reproduce behaviors comparable to those observed at smaller δ in the base model, while the distributional properties remain essentially unchanged for analogous *B* conditions.

### Appendix A.2. Non-Instantaneous Translation

It has also been recognized that assuming instantaneous translation represents another strong idealization, since translation relies on a complex molecular machinery.

Within our framework, this leads to the following stochastic delay-differential system:

(A6) $$\begin{array}{cc} \frac{dx}{dt} & =\delta \left(\frac{1+\gamma_{2}y^{2}}{\beta_{0}+1+\gamma_{2}y^{2}}-x\right) \end{array}$$

(A7) $$\begin{array}{cc} \frac{dy}{dt} & =w-(d+\nu (t))y \end{array}$$

(A8) $$\begin{array}{cc} w(t) & =\int_{0}^{\infty }K_{x}\left(s\right)x\left(t-s\right)\;ds \end{array}$$

where w(t) denotes a delayed version of x(t) induced by the kernel $K_{x}\left(s\right)$.

Assuming an acquisition-fading kernel, the system admits a finite- dimensional representation:

(A9) $$\begin{array}{cc} \frac{dw_{1}}{dt} & =b_{1}\left(x-w_{1}\right) \end{array}$$

(A10) $$\begin{array}{cc} \frac{dw}{dt} & =b_{2}\left(w_{1}-w\right) \end{array}$$

As in the delayed-feedback scenario, we reproduce the simulations of the base model to highlight possible differences. As previously, we restrict the analysis to the quantities related to y(T). For the newly introduced kernel parameters, we fix the characteristic delay time to $\tau_{c}=1$, which leads to the choice $b_{1}=b_{2}=2$. The resulting curves for $\langle Y\rangle ,Y_{max},Y_{min}$ are shown in Figure A3.

Comparing these results with the previous ones, we observe that introducing a delay in x(t) produces effects closely analogous to those generated by delaying y(t). Since identical characteristic times are employed, the two delays appear essentially equivalent in terms of their impact on the behavior of y(T). Consequently, the conclusions mirror those obtained for delayed TF feedback. For this reason, we do not further analyze the probability density functions for different *B* values: once suitably adjusted, their shapes remain consistent with those of both the base model and the delayed-feedback variant, as discussed above.

### Appendix A.3. Combining the Two Delays’ Effects

The simultaneous presence of both the delay mechanisms introduced in the two previous sections leads to the following extended model:

(A11) $$\begin{array}{cc} \frac{dx}{dt} & =\delta \left(\frac{1+\gamma_{2}z^{2}}{\beta_{0}+1+\gamma_{2}z^{2}}-x\right) \end{array}$$

(A12) $$\begin{array}{cc} \frac{dy}{dt} & =w-(d+\nu (t))y \end{array}$$

(A13) $$\begin{array}{cc} w(t) & =\int_{0}^{\infty }K_{x}\left(s\right)x\left(t-s\right)\;ds \end{array}$$

(A14) $$\begin{array}{cc} z(t) & =\int_{0}^{\infty }K_{y}\left(s\right)y\left(t-s\right)\;ds \end{array}$$

When both delays are present, one may reasonably expect the anticipatory effect in the dynamics to be reinforced—perhaps not as a simple superposition of the two contributions, but without generating qualitatively new regimes. This expectation is confirmed by the behavior of $\langle Y\rangle ,Y_{max},Y_{min}$ shown in Figure A4.

Beyond the overall stronger “anticipatory” effect, this configuration reveals an additional noteworthy feature. Although the δ values remain unchanged, the delay shifts the peak of the curve further to the right. A similar tendency was already visible in the previous case, but here, the shift appears significantly more pronounced and clearly defined.
